# Physical activities aid in tumor prevention: A finite element study of bio-heat transfer in healthy and malignant breast tissues

**DOI:** 10.1016/j.heliyon.2024.e34650

**Published:** 2024-07-15

**Authors:** Mohammad Junaid, Abul Mukid Mohammad Mukaddes, Md. Mahmud-Or-Rashid

**Affiliations:** a Department of Mechanical Engineering, Shahjalal University of Science and Technology, Sylhet, 3114, Bangladesh; b Department of Industrial and Production Engineering, Shahjalal University of Science and Technology, Sylhet, 3114, Bangladesh

**Keywords:** Breast cancer, Tumor prevention, Bio-heat, Finite element method, Exercise intensity, Metabolic heat, Blood perfusion, Hyperthermia, Temperature distribution

## Abstract

The objective of the present research is to explore the temperature diffusion in healthy and cancerous tissues, with a specific focus on how physical activity impacts on the weakening of breast tumors. Previous research lacked numerical analysis regarding the effectiveness of physical activity in tumor prevention or attenuation, prompting an investigation into the mechanism behind physical activity and tumor prevention from a bio-heat transfer perspective. The study employs a realistic model of human breasts and tumors in COMSOL Multiphysics® to analyze temperature distribution by utilizing Penne's bio-heat equation. The research examines their influence on tissue temperature by varying tumor diameter (10–20 mm) and exercise intensities (such as walking speeds and other activities like carpentry, swimming, and marathon running). Results demonstrate that cancerous tissues generate notably more heat than normal tissues at rest and during physical activity. Smaller tumors exhibit higher temperatures during exercise, emphasizing the significance of tumor size in treatment effectiveness. Tumor temperatures range between 40 and 43.2 °C, while healthy tissue temperatures remain below 41 °C during physical activity. High-intensity exercises, particularly swimming, walking at 1.8 m/s, and marathon running, display a therapeutic effect on tumors, increasing effectiveness with intensity. The temperatures of healthy and malignant tissues rise noticeably due to constant metabolic heat and decreased blood flow. The study also identifies the optimal duration of high-intensity exercise, recommending at least 20 min for optimal therapeutic outcomes. The outcomes of this research would help individuals, doctors, and cancer researchers understand and weaken malignant tissues.

## 1 Introduction

Breast cancer is as one of the widespread types of cancer affecting females globally, with significant implications for health, well-being, and longevity. It arises from the uncontrolled growth of cells within the breast tissue, often forming a lump or mass that can lay out to different organs of the human body if left untreated [1]. The World Health Organization (WHO) declared that breast cancer is the most frequent cancer worldwide, both in poor and rich regions, making it a critical health concern across diverse populations [2]. Breast cancer encompasses various subtypes, each with its distinct characteristics and prognoses. The most general types include ductal carcinoma in situ (DCIS), invasive ductal carcinoma (IDC), and invasive lobular carcinoma (ILC) [3]. While genetic factors, for instance mutations in the BRCA1 and BRCA2 genes contribute to some cases, environmental and lifestyle factors also play significant roles in the evolution of breast cancer [4]. Early detection through screening methods such as mammography has substantially improved outcomes for breast cancer patients by enabling the diagnosis of tumors at an earlier, more treatable stage [5]. Treatment approaches for breast cancer typically involve a combination of surgery, radiation therapy, chemotherapy, targeted therapy, hormone therapy, or a combination of these modalities, depending on the classification and phase of the cancer [6]. Surgery remains a keystone in the handling of breast cancer, with options ranging from lumpectomy or mastectomy for localized disease to more extensive procedures for advanced cases [7]. Chemotherapy deploys medicines to destroy cancer cells or resist their growth and is often administered before or after surgery to reduce tumor size or prevent recurrence [8]. Radiation therapy targets localized cancer cells with high-energy beams, reducing the possibility of cancer deterioration in the breast after surgery [9]. Hormone therapy, also known as endocrine therapy, is particularly effective in hormone receptor-positive breast cancers by blocking the hormones that fuel tumor growth or reducing the body's estrogen levels [10]. Targeted therapy drugs, such as trastuzumab (Herceptin), work against specific molecular targets, such as the HER2 protein, found in some breast cancers, leading to more tailored and effective treatments [11]. Thermal therapies for breast tumors encompass various techniques aimed at applying heat to the affected tissue to induce tumor destruction. One such method is radiofrequency ablation (RFA), which employs high-pitch electricity to generate heat energy and devastate cancerous cells. RFA is effective in treating small breast tumors, particularly in patients who are not suitable candidates for surgery or those seeking minimally invasive alternatives [12]. Another thermal therapy modality utilized in the treatment of breast tumors is laser-induced thermotherapy (LITT). LITT involves inserting a thin fiber optic probe into the tumor, through which laser energy is emitted to heat and ablate the cancerous tissue. This technique offers precise tumor targeting and minimal invasiveness, making it a viable option for selected cases of breast cancer [13].

Hyperthermia, a therapeutic approach involving the application of heat to elevate tissue temperature, has also been explored for the treatment of breast tumors. By subjecting cancerous cells to temperatures typically ranging from 40 °C to 45 °C, hyperthermia can enhance the effectiveness of other treatment modalities such as chemotherapy and radiation therapy. This synergistic effect arises from increased blood flow, improved drug delivery, and enhanced radiation sensitivity within the tumor microenvironment [14]. As a separate therapeutic target variable, tissue temperature has drawn more clinical attention. Numerous common human disorders are significantly influenced by tissue temperature. This generally means that studies that comprehend the fundamental mechanisms regulating tissue temperature are essential for creating sensible, efficient, and safe clinical therapies for cancer patients [15]. One of the major goals of the thermal simulation of essential tissues is the detection of malignant tumors. On the other hand, malignant cells have a higher metabolism than healthy ones. As a result, it is a known fact that malignant tissues typically have higher temperatures than healthy tissues [16].

The geometric modeling and thermal simulation of tissue heavily rely on thermal analysis. Alamouti et al. [17] discovered the effects of tumor growth on the temperature distribution in three diameters of tumors, namely, 10, 15, and 20 mm, and they observed that the tumor center produces a higher temperature than its surrounding healthy tissue. They found that a decrease in the blood perfusion does not enhance the tumor temperature significantly, but changing the values of the tumor metabolism affects its temperature significantly. Physical activity has emerged as a crucial aspect of breast cancer management, encompassing various forms of exercise, for instance aerobic activities, strength training, yoga, and pliability workouts. Research has shown the multifaceted advantages of regular physical workout for breast cancer patients, from diminishing the chance of developing the disease to improving treatment outcomes and enhancing overall well-being of life [18]. Epidemiological studies consistently indicate that engaging in regular physical activity is interconnected with a lesser possibility of causing breast cancer [19]. Physical activity exerts its protective effects through mechanisms such as reducing circulating levels of estrogen and insulin, which are known to promote breast cancer development [20]. Physical workout plays a complementary role in enhancing the effectiveness of breast cancer treatments. Exercise has been proved to mitigate the adverse aftereffects of chemotherapy, and radiation therapy, including pain, fatigue, nausea, and loss of muscle mass [21]. Moreover, regular exercise can help maintain cardiovascular health and preserve bone density, which may be compromised during certain cancer treatments [22]. Beyond its physiological benefits, physical activity contributes to the psychosocial well-being of breast cancer survivors. Engaging in regular exercise can alleviate symptoms of depression and anxiety, improve self-regard, and foster a perception of control and empowerment over one's health [23]. Additionally, participation in group-based exercise programs provides social support and camaraderie, mitigating feelings of isolation often experienced during cancer treatment and recovery [24]. Physical activity lowers cancer occurrence and the likelihood of reappearance and ensures longer, higher-quality lives for patients with disseminated illnesses, according to research by Hojman et al. [25]. Additionally, exercise reduces tumor growth in all phases of tumor development and across all cancer histologies. According to Hofmann [26], resistance training and moderate-to-vigorous aerobic exercise have both been found to have overall positive impacts on cancer prevention and treatment. According to Dethlefsen et al. [27], systemic modifications to a 2-h workout reduced breast cancer viability, whereas adaptations to a 6-month training regimen had no effect. A complementary anticancer therapy strategy is proposed by Idorn and Hojman [28] depending on the effects of NK cell mobilization and activation, in addition to changes in blood perfusion and body temperature caused by workouts. According to Ref. [29], the metabolic heat generated during running can alter with factors such as intensity, duration, environmental conditions, and individual physiology. However, as a general estimate, research suggests that during running, the metabolic heat production can range from around 15 to 20 times the resting metabolic rate, or METs (metabolic equivalents), where 1 MET is equivalent to the resting metabolic rate. According to Ref. [30], the increase in metabolic heat generation during physical workouts could fluctuate with factors such as the intensity and duration of the activity, in addition to individual differences such as fitness level and body composition. In order to produce energy for the contraction of skeletal muscles, muscular exercise raises metabolism by five to fifteen times the resting rate. However, as a general estimate, physical activity can increase metabolic heat generation from two to twenty times above the resting metabolic rate or even higher during intense activities [31]. According to Betof et al. [32], acute exercise can increase blood flow to tumors, potentially leading to increased metabolic heat production within the tumor microenvironment. According to these results, the metabolic rate and the blood perfusion of tumor also increase linearly with exercise intensities. For the workout scenario, Paul et al. [33] created a realistic human body geometry, and the thermal response was computed for three different exercise intensities—walking on a treadmill at 0.9, 1.2, or 1.8 m/s. Over the course of 5 min, the muscle's metabolic rate was allowed to rise linearly from its resting condition to its peak metabolic heat generating rate for the appropriate exercise intensity. The local blood perfusion rate was considered to be directly proportional to increases in the metabolic heat generation rate. At resting conditions, the metabolic heat rate and blood perfusion rate are 553.5 $W/m^{3}$ and 0.0005 $s^{-1}$ inside the muscle, respectively. Metabolic heat rates of 0.9, 1.2, and 1.8 m/s walking speeds are 7.23, 9.7, and 13.73 times the resting condition, respectively, and the blood perfusion rates are 7.23, 9.7, and 13.73, respectively, inside the muscle. The metabolic heat rates for marathon running, carpentering, and swimming are 7918, 4198.08, 6598.79 $W/m^{3}$, respectively, according to two more investigations by JVGA [34] and Shrestha et al. [35]. These rates are 7.585, 11.922, and 14.31 times higher than the resting condition. However, not all the existing studies provide a clear explanation of how exercise prevents tumor growth. Since there is no evidence as to why this mechanism occurs, a numerical investigation is required to understand its main reason. Also, all the previous studies lacked quantitative analysis on the effectiveness of physical activity in reducing or preventing tumors, so we were inspired to explore the relationship between physical activity and tumor prevention. Our approach focused on examining this correlation from a perspective rooted in bio-heat transfer principles. Tissues and tumors are susceptible to temperature changes. One may be able to do several analyses at various sites of the tissues, including the impacts of temperature fluctuations, using a mathematical simulation built on the finite element approach. The present scientific work offers a practical mathematical model of the tissue and the tumor. The model was developed using commercial software, COMSOL Multiphysics® [36]. Finite element analysis's fundamental governing equation was Penne's bio-heat equation [37]. The model is simulated using COMSOL Multiphysics® since Penne's bio-heat equation, which is employed in this tool, produces findings that are almost identical to those of the experiments [38]. This research delves into exploring the heat production in cancerous tissues and healthy tissues during rest and physical activity periods. It examines the correlation between physical activity and the potential prevention of tumor development and evaluates the influence of tumor size and growth. Additionally, the study investigates whether engaging in physical exercises can function as a form of thermal therapy to deter breast tumors. Furthermore, it analyzes the impacts of workout intensity and duration on achieving the most effective outcomes in thwarting breast tumors, considering the principles of bio-heat transfer.

## 2 Materials and method

### 2.1 Flow of analysis

Using Penne's bio-heat equation, 3D finite element models of human muscle and tumors were originated to inquire into the temperature diffusion at different physical activities. The tumor and its response to heat distribution were observed, following the efficacy of different hyperthermal treatments, and then it was determined whether the physical exercises work as conventional hyperthermal treatments for tumors. The model is simulated using COMSOL Multiphysics® since Penne's bio-heat equation, which is employed in this tool, produces findings that are almost identical to those of the experiments [38]. Graphs are plotted in OriginePro-2024 [39] by extracting the results from COMSOL Multiphysics®.

### 2.2 Model geometry

**Fig. 1:**
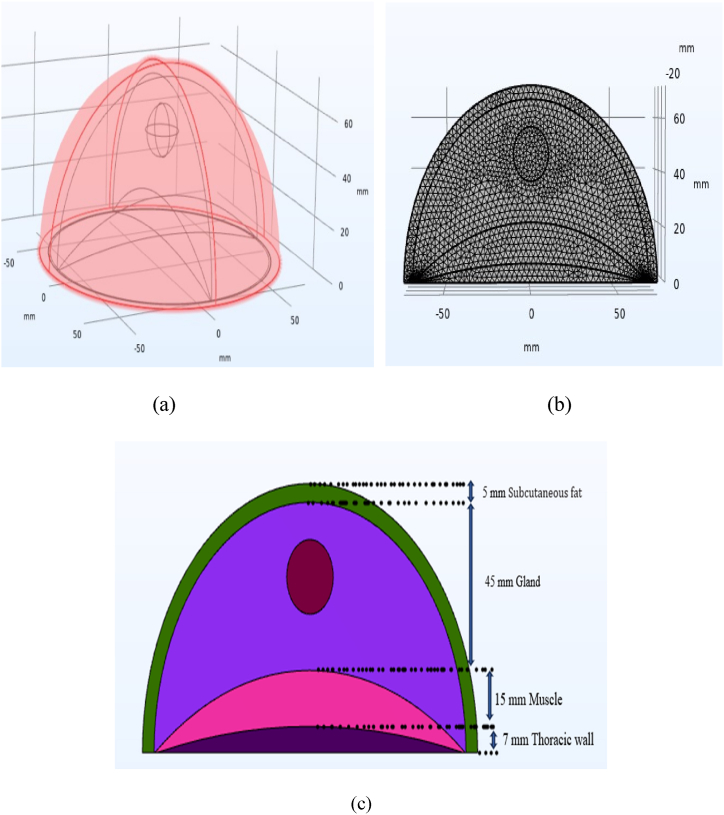
(a) A 3-D model of the breast featuring a spherical tumor, (b) finite element mesh illustrating a cross-section of the breast and tumor, and (c) the measurements of various layers [40].

Fig. 1(a) and (b) depict the configuration of the breast and tumor, along with their finite element mesh. Notably, the breast surface is devoid of any clothing. Spherical tumors, varying in diameter from 10 to 20 mm, were positioned either 49.5 mm above the base surface or 22.5 mm below the areola along the vertical centerline of the breast. This positioning aligns with the entire height of the areola area, which is situated 72 mm away from the ribcage [40]. The measurements of the different layers of the breast are outlined in Fig. 1(c). To simplify computational analysis and enhance clarity in observing temperature distribution, the full model was partitioned into two sections.

### 2.3 Governing equation

The primary equation used for calculating the temperature distribution within human tissue is known as Pennes' bio-heat equation [37,41,42]. COMSOL Multiphysics® is used to simulate the model since Penne's bio-heat equation is used in this tool and gives nearly similar results to that of the experimental ones [38,42,43].

To resolve the governing equation (1) the finite element approach was used. The weighted residual and the Crank-Nicolson method were applied to for space and time discretization, respectively. Equations used in this study are incorporated in COMSOL Multiphysics® which are as follows [[42], [43], [44]]:

(1) $$\rho c_{p}\frac{\partial T}{\partial t}+\nabla .q=\rho_{b}c_{b}\omega_{b}\left(T_{b}-T\right)+Q_{m}+Q$$

(2) q=−k.∇T

Where, $c_{p}$ is the specific heat (J/kg °C), ρ is the density of the tissue (kg/m^3^), $\rho_{b}$ denotes the density of the blood (kg/m^3^), $c_{b}$ denotes the specific heat of the blood (J/kg °C), $\omega_{b}$ is the blood perfusion rate (1/s), $T_{b}$ is the known arterial temperature (°C), T is the unknown tissue temperature (°C), t is time (min), q is the heat flux ($W/m^{2}$), k is the thermal conductivity of the tissue (W/m °C), ${\;Q}_{m}$, is the volumetric metabolic heat (W/m^3^), and Q is the external heat source (W/m^3^). Here, no external heat is applied, so Q is assumed to be zero.

### 2.4 Physiological properties

#### 2.4.1 Physiological properties of breast tissues and tumors

The breast is a collection of different layers. The layers are subcutaneous fat, gland, muscle, and thoracic wall, which have their respective properties. Physiological properties of various layers and tumors are displayed in Table 1.

**Table 1:** Physiological properties of different layers of breast tissue and tumor [[33], [34], [35],^40^,[45], [46], [47], [48]].

**Tissues**	k[W/(m°C)]	$\rho \;\left[kg/m^{3}\right]$	$c_{p}\;\left[J/\left(kg{}^{\circ}C\right)\right]$
**Subcutaneous fat**	0.21	930	2770
**Gland**	0.48	1050	3770
**Muscle**	0.48	1100	3800
**Thoracic wall**	0.48	1100	3800
**Tumor**	0.565	1027.4	3600

According to Ref. [29], the metabolic heat generated during running alters with factors like intensity, duration, environmental conditions, and individual physiology. However, as a general estimate, research suggests that during running, the metabolic heat production can range from around 15 to 20 times the resting metabolic rate, METs (metabolic equivalents), where 1 MET is equivalent to the resting metabolic rate. According to Ref. [30], the increase in metabolic heat generation during physical activities alters with changing factors like the intensity and duration of the activity, as well as individual differences such as fitness level and body composition. To produce energy for the contraction of skeletal muscles, muscular exercise raises metabolism by five to fifteen times the resting rate. However, as a general estimate, physical activity can increase metabolic heat generation anywhere from two to twenty times above the resting metabolic rate, or even higher during very intense activities. A study by Paul et al. [33] found that under resting conditions, the metabolic heat and blood perfusion inside the muscle are 553.5 $W/m^{3}$ and 0.0005 $s^{-1}$, respectively. As walking speeds increase to 0.9, 1.2, and 1.8 m/s, the corresponding metabolic heat rates become 7.23, 9.7, and 13.73 times the resting condition, while blood perfusion rates are 7.2, 9.6, and 13.8 times, respectively. The study suggests a linear increase in the breast's metabolic heat and blood perfusion with exercise intensities, with local blood perfusion rate directly proportional to metabolic heat generation rate increments. Another study by JVGA [34] and Shrestha et al. [35] indicated that the metabolic heat rates for carpentry, swimming, and marathon (running) are 4198.08, 6598.79, and 7918 $W/m^{3}$, respectively. These rates are 7.585, 11.922, and 14.31 times higher than the resting condition, respectively. According to Betof et al. [32], acute exercise can increase blood flow to tumors, potentially leading to increased metabolic heat production within the tumor microenvironment. Based on these studies, it is assumed that the metabolic heat and blood perfusion rates of the breast and tumor increase linearly at the same rate as the muscle with exercise intensities. The study presents the corresponding metabolic heat and blood perfusion rates for different exercises in Table 2.

**Table 2:** Metabolic heat and blood perfusion of the breast tissue at different activity levels [31,[33], [34], [35],^40^,[45], [46], [47]].

**Activities**	$Q_{m}\;\left[W/m^{3}\right]$	$\omega_{b}\;\left[s^{-1}\right]$
**Resting**	700	0.00045
**1.8 m/s walking speed**	9611	0.00617
**1.2 m/s walking speed**	6790	0.00436
**0.9 m/s walking speed**	5061	0.00325
**Carpentry**	5309.5	0.0034
**Swimming**	8345.4	0.0053
**Marathon Running**	10017	0.0064

#### 2.4.2 Metabolic heat and blood perfusion in tumors of varying sizes during different physical activities

Tumor properties are not found directly in the literature. Researchers use tumor volume doubling time to compute the tumor metabolic heat generation. The following equation was used by Ng and Sudharsan [40] and Camilleri [45]:

(3) $$q_{m}\tau =C$$

where $q_{m}$ ($W/m^{3}$) is the metabolic heat production, τ (days) is the tumor volume doubling time, and C is a constant equal to 3.27 × 106 $W.\;\text{Day}\;/\;m^{3}$. Ng and Sudharsan [40] estimated the tumor volume doubling time from the diameter of the tumor and using equation (4) where D is the diameter.

(4) $$D=\exp\;\left[0.002134\left(\tau \;-\;50\right)\right]\times 10^{-2}\text{.}$$

According to Camilleri [45], the blood perfusion rate in tumors varies between 0.00129 and 0.005 $s^{-1}$, while for normal breasts, it ranges from 0.00045 to 0.00093 $s^{-1}$. Building on the findings of a previous study [33], it was anticipated that tumors' blood perfusion and metabolic heat would both rise linearly with increasing exercise intensities at roughly the same rates. Utilizing equations (3), (4)) along with the data from Ref. [45], we computed the metabolic heat for tumors of various diameters (10, 12.5, 15, 17.5, and 20 mm) under resting conditions. Subsequently, we calculated the metabolic heat for these tumors under different exercise intensities, along with their corresponding blood perfusion rates, as presented in Table 3.

**Table 3:** Physiological properties of tumors of different sizes for different activities [[33], [34], [35],^40^,[45], [46], [47], [48]].

Activities		10 mm	12.5 mm	15 mm	17.5 mm	20 mm
Resting	$Q_{m}\;[W/m^{3}$	65400	21156.83	13625	10473	8724.42
	$\omega_{b}\;\left[s^{-1}\right]$	0.003	0.0015	0.0014	0.00133	0.00129
1.8 m/s walking speed	$Q_{m}\;[W/m^{3}$]	897942	290483.3	187071.25	143794.3	119786.3
	$\omega_{b}\;\left[s^{-1}\right]$	0.041	0.0133	0.0086	0.0072	0.0069
1.2 m/s walking speed)	$Q_{m}\;\left[W/m^{3}\right]$	634380	205221.25	132162.5	101588.1	84626.88
	$\omega_{b}\;\left[s^{-1}\right]$	0.029	0.0094	0.00605	0.00514	0.0048
0.9 m/s walking speed	$Q_{m}\left[W/m^{3}\right]$	472842	152963.88	98508.75	75720	63077.6
	$\omega_{b}\;\left[s^{-1}\right]$	0.0216	0.00701	0.0045	0.0038	0.0035
Carpentry	$Q_{m}\;\left[W/m^{3}\right]$	496059	160474.6	103345.63	79437.7	66175
	$\omega_{b}\;\left[s^{-1}\right]$	0.0227	0.00735	0.0047	0.004	0.0036
Swimming	$Q_{m}\;\left[W/m^{3}\right]$	779699	252232	162437.25	124859.2	104012.6
	$\omega_{b}\;\left[s^{-1}\right]$	0.035	0.011	0.0074	0.0063	0.0057
Marathon running	$Q_{m}\;\left[W/m^{3}\right]$	935874	302754.3	194974	149869	124846.5
	$\omega_{b}\;\left[s^{-1}\right]$	0.042	0.013	0.0089	0.0075	0.0068

### 2.5 Boundary conditions

We make the assumption that the beginning condition in this investigation is normothermic. When the body and its environment achieve temperature balance, this equilibrium happens. It was assumed that the skin's outer layer was exposed to a temperature of 21 °C, with a total heat transfer coefficient (h) of 13.5 $W/m^{2}{}^{\circ}C$ [40]. As the starting temperature of human muscle, 37 °C was established as the body's core temperature, encompassing the breasts and muscles [41]. Based on the findings of Alamouti et al. [17] and Bousselham et al. [48], the temperature at the tumor's center was approximately 1.5 °C higher than the surrounding healthy tissues. Therefore, for this study, we adopted an average initial temperature of 38.5 °C for the tumor under normal conditions. Three key mechanisms—convection, radiation, and evaporation—transfer heat between body and surroundings. As part of the defined boundary conditions for environmental exposure, we integrate the convection, radiation, and evaporation mechanisms in our analysis to obtain a net heat transfer coefficient (h). In COMSOL Multiphysics®, the boundary condition is represented by the following equation [42,44]:

(5) $$q_{o}=h\;\left(T_{ext}-T\right)$$

In this case, T is the unknown tissue temperature (°C), h is the total heat transfer coefficient (W/m^2^ °C), $q_{o}$ is the heat flux between body and environment (W/m °C), and $T_{ext}$ is the ambient temperature (°C).

### 2.6 Grid independent test

**Fig. 2:**
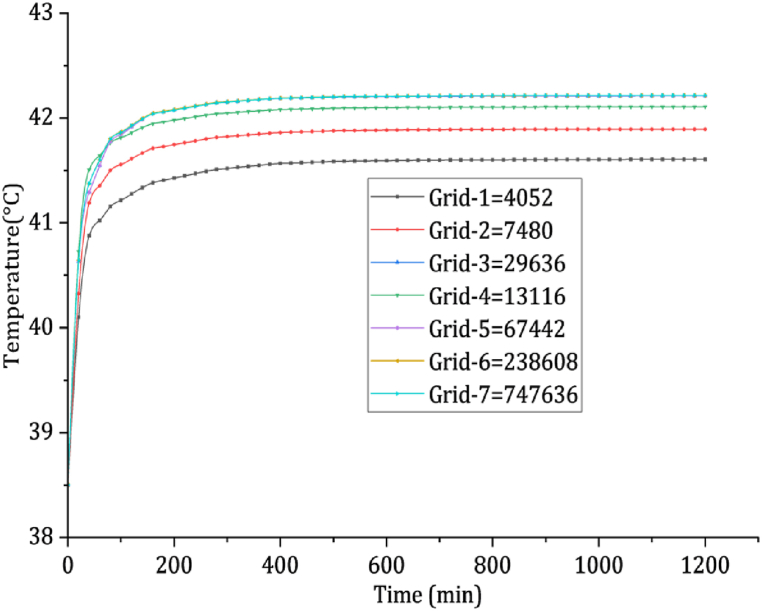
Compares different numbers of elements and shows the temperature generated in the center of a tumor of 10 mm diameter while walking at a speed of 1.2 m/s.

A grid independence test determines the most appropriate mesh that balances computational efficiency with result accuracy. Seven different grid elements were employed to assess mesh sensitivity, incorporating wall refinement to observe the impact of element size on computational outcomes. The focus is comparing the temperature generation at the core of a tumor of 10 mm diameter during a walking speed of 1.2 m/s. Fig. 2 illustrates the temperature variation over time for various numbers of elements. The analysis indicates that Grids 4 through 7 yield consistent results. Grid-5 is chosen for its optimal balance of computational speed and numerical precision for subsequent investigations.

## 3 Results and discussion

### 3.1 Model Validation-1

Detecting cancerous tumors is a primary goal when simulating the thermal behavior of vital tissues. Cancer cells typically have a higher metabolism than healthy cells, resulting in higher temperatures in cancerous tissues. Bousselham et al. [49] conducted a study on brain tumors, revealing that the maximal temperature increase at the tumor's core is approximately 1.5 °C higher than in normal tissue. Similarly, Alamouti et al. [17] and Bousselham et al. [48] observed a temperature difference of around 1.5 °C between the core of the tumor and the neighboring healthy tissues. Lahiri et al. [50] demonstrated through experimentation and computation that breast tumors and their surrounding regions experience the highest temperature rise. Rashmi et al. [51] further confirmed that tumors, regardless of size, elevate local temperatures, with increased metabolic heat generation intensifying the effect.

**Fig. 3:**
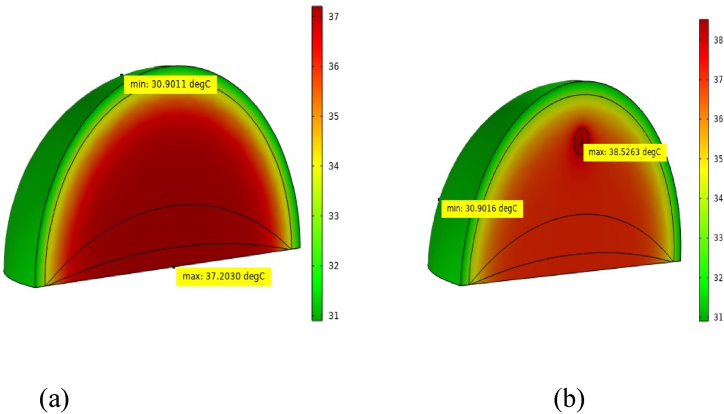
Temperature diffusion in breast at resting condition: (a) healthy breast tissue and (b) malignant breast tissue.

Fig. 3(a) depicts a normal human breast after 30 min of rest. The highest recorded temperature is 37.2 °C at the bottom of the thoracic wall. Conversely, Fig. 3(b) illustrates a human breast with a 10 mm tumor positioned 22.5 mm below the skin surface or 49.5 mm above the ribcage after the same rest period. In this scenario, the maximal temperature recorded at the tumor's core reaches 38.53 °C, consistent with previous findings [17,49]. The temperature disparity found in our study between healthy and malignant breast tissues ranges from 1.33 to 1.5 °C, which satisfies all studies mentioned above.

### 3.2 Model Validation-2

**Fig. 4:**
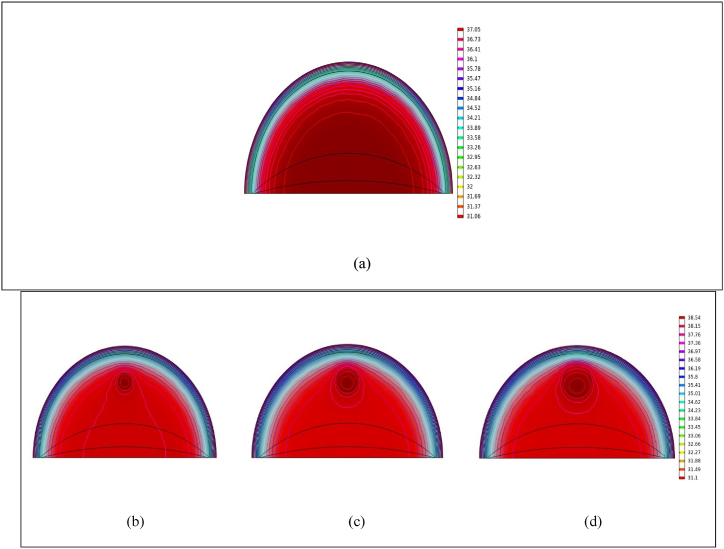
Shows the temperature distributions in a healthy breast and in breasts with malignant tumors at rest. Specifically, panel (a) displays the temperature contour of a normal breast, while panels (b), (c), and (d) depict the temperature contours of breasts with tumors measuring 10, 15, and 20 mm, respectively.

Fig. 4(a) displays temperature contours representing a healthy breast at rest for 30 min. In contrast, Fig. 4(b), (c), and (d) depict the temperature contours of breasts hosting tumors of different diameters: 10, 15, and 20 mm, respectively, under identical conditions to the healthy breast. It's worth noting that initially, the healthy breast shows the largest cold area. As the tumor size increases, this cold area gradually shrinks concentrically. This consistent reduction indicates either a decrease in cold regions or an increase in warm areas within the breast. A notable observation is that the healthy breast exhibits the most significant cold area. The breast with a 15 mm tumor displays a wider high-temperature region between breasts with 10 mm and 15 mm tumors. Similarly, the breast with a 20 mm tumor shows a broader high-temperature region than the breast with a 15 mm tumor. This trend aligns with the findings of Alamouti et al. [17], Ng and Sudharsan [40], and Bousselham et al. [49] in their respective studies.

### 3.3 Temperature distribution during exercise in the breasts with and without tumors

**Fig. 5:**
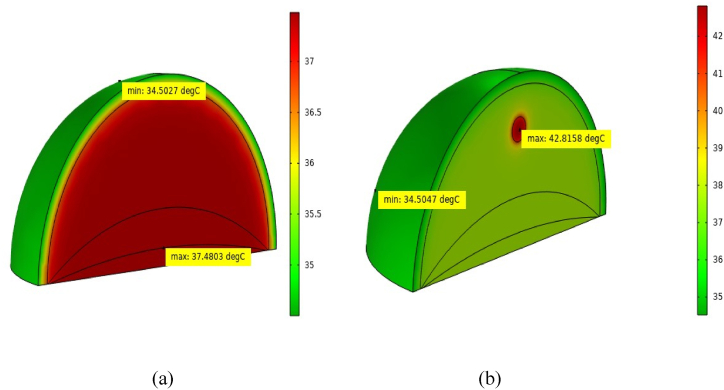
Illustrates the temperature distribution at a 1.8 m/s walking speed, comparing (a) a healthy breast and (b) a malignant breast containing a 10 mm tumor.

Fig. 5(a) portrays the conditions of a normal human breast following 30 min of walking at a 1.8 m/s speed. In this scenario, the highest temperature recorded at the thoracic wall and muscle interface is about 37.48 °C. Fig. 5(b) portrays the conditions of a human breast containing a 10 mm tumor positioned 22.5 mm below the skin surface or 49.5 mm above the ribcage at the same condition. In this scenario, the maximal temperature recorded at the core of the tumor reaches 42.82 °C. In walking conditions, the highest temperature is found at the center of the malignant breast, and it is also found in resting conditions. The temperature difference between healthy and malignant breasts is about 5.35 °C.

### *3.4* Analysis of temperature distribution in breast and tumors at rest and physical exercise

**Fig. 6:**
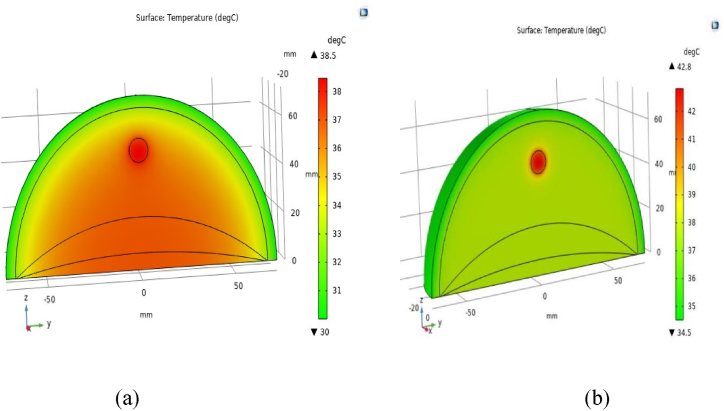
Demonstrate the temperature diffusion in a female breast with a 10 mm diameter tumor placed 22.5 mm below the skin surface: (a) while at rest, and (b) during walking at a 1.8 m/s speed.

The human body relies on a complex thermoregulation system to maintain its internal temperature, a critical function for overall health and well-being. There are two primary factors: metabolic heat generation ($Q_{m}$) and blood perfusion ($\omega_{b}$). Metabolic heat, produced by various biochemical processes within the body, serves to elevate its temperature, while blood perfusion facilitates the distribution of heat, working to regulate the core temperature by dispersing it throughout the body. When metabolic heat increases, the core temperature rises, whereas an increase in blood perfusion tends to have the opposite effect, aiding in lowering the core temperature. These two factors are intricately linked and are influenced by physical activity, with variations occurring concurrently but exerting opposing effects on body temperature regulation. Among these factors, metabolic heat plays a dominant role, particularly during periods of intense physical activity, leading to a notable elevation in core temperature. This relationship between metabolic heat generation, blood perfusion rate, and physical activity is illustrated in Fig. 6(a), which portrays the conditions of a female breast containing a 10 mm tumor positioned 22.5 mm below the skin surface or 49.5 mm above the ribcage following 30 min of rest. In this scenario, the highest temperature recorded at the core of the tumor reaches 38.5 °C, aligning with previous research findings concerning brain tumors [17,40,49].

Transitioning to Fig. 6(b), the scenario shifts to a human breast with a 10 mm tumor after 30 min of walking on a treadmill at a 1.8 m/s speed. Here, the overall temperature of the breast increases notably, with the highest temperature observed at the tumor's core reaching approximately 42.82 °C. This increase underscores the significant heat generated by cancerous tissues or tumors compared to surrounding normal tissues, evident both during periods of rest and physical activity. This heightened heat generation can be attributed to elevated levels of both metabolic heat generation and blood perfusion rate within the tumors.

This intricate interplay of metabolic blood perfusion, heat generation, and physical activity underscores the dynamic nature of thermoregulation within the human body. Understanding these mechanisms sheds light on the body's remarkable capability to control thermal equilibrium and holds implications for various medical conditions, particularly those involving abnormal heat generation, such as tumors.

### 3.5 The impact of tumor size on temperature distribution within healthy tissues and tumors during physical exercise

**Fig. 7:**
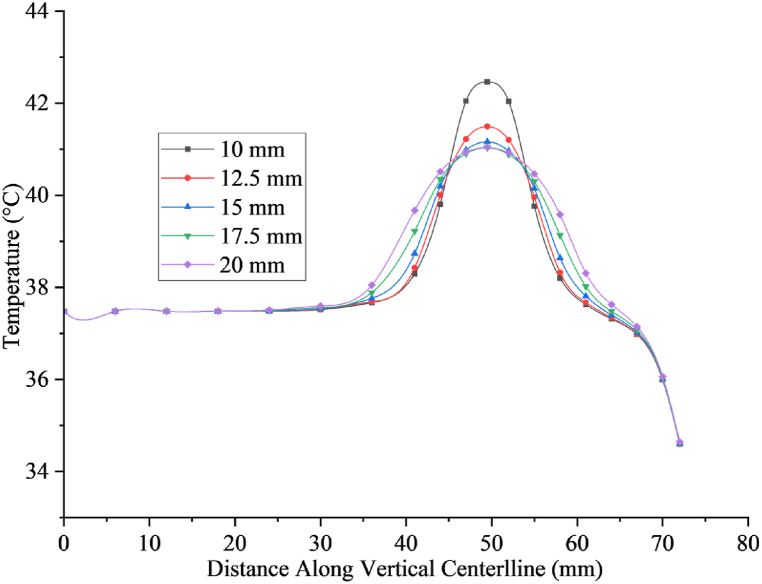
Illustrates how temperature varies along the centerline of the tumor, stretching from the rib to the skin, for different tumor sizes when walking at a speed of 1.8 m per second for 30 min.

Fig. 7 illustrates the temperature distribution within the breast tissue hosting tumors of varying sizes, precisely 10, 12.5, 15, 17.5, and 20 mm in diameter, positioned 22.5 mm beneath the skin surface after a 30-min treadmill session at 1.8 m/s. Temperature profiles were plotted along the tumor's and the breast's central vertical axis, extending from the ribcage to the areola region. Notably, smaller tumors, such as those with a 10 mm and 12.5 mm diameter, exhibit relatively smaller high-temperature zones than larger tumors, like those measuring 17.5 mm and 20 mm. This indicates a trend where, as tumor diameter increases, there is an analogous homocentric contraction of the cold area in the breast. These findings are consistent with observations made under resting conditions, as documented in Refs. [17,40,49].

**Fig. 8:**
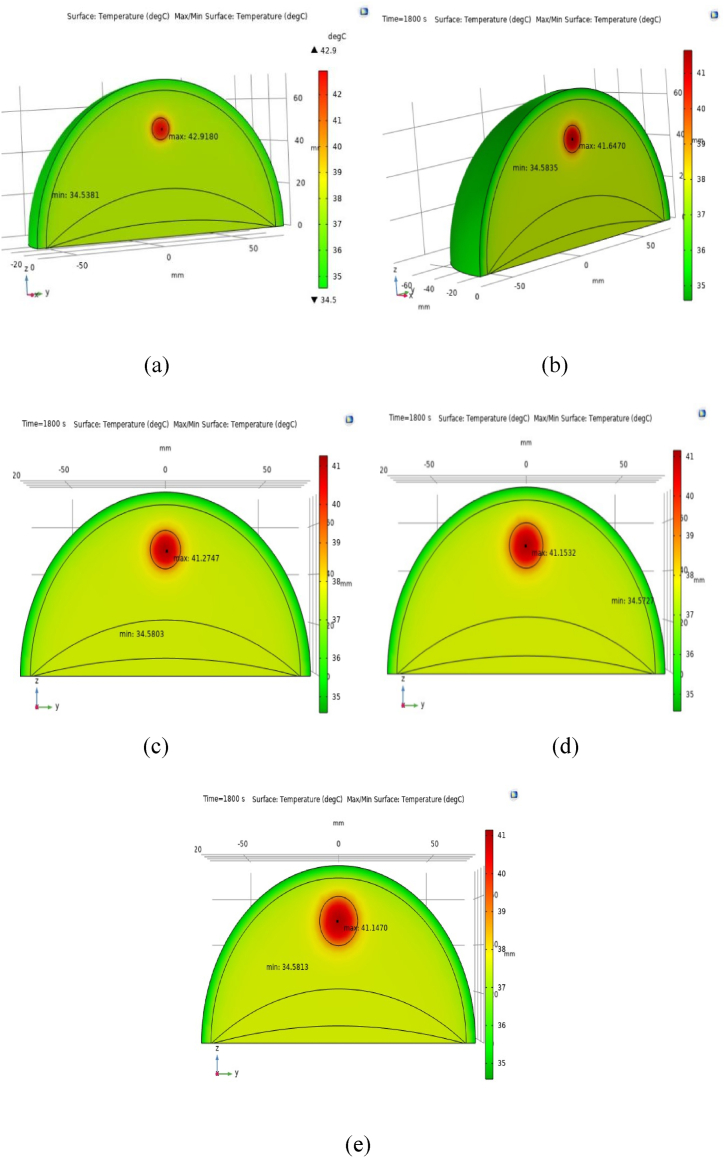
(a), (b), (c), (d), and (e) illustrate the temperature distributions in breast tissues with tumors measuring 10, 12.5, 15, 17.5, and 20 mm in diameter, respectively, during marathon running.

Fig. 8(a), (b), (c), (d), and (e) illustrate the temperature distributions in breast tissues with tumors measuring 10, 12.5, 15, 17.5, and 20 mm in diameter, respectively, during marathon running. The central temperatures induced in tumors of 10, 12.5, 15, 17.5, and 20 mm diameters are recorded as 42.92 °C, 41.65 °C, 41.28 °C, 41.15 °C, and 41.14 °C, respectively. Intriguingly, the 10 mm tumor generates the highest temperature during the specified physical activity despite its smaller size. This suggests smaller tumors generate more heat than larger ones during physical activity. Comparative analysis with Fig. 7, Fig. 8 underscores that tumors with smaller diameters exhibit elevated temperatures at their centers during both walking (1.8 m/s) and marathon running, attributed to heightened metabolic heat and blood perfusion. These results emphasize the significant influence of tumor volume on temperature allocation within cancerous tissues. Specifically, smaller tumors dominate metabolic heat production, which becomes more apparent during physical activity. Therefore, understanding the connection between tumor volume and heat generation is crucial for assessing tumor behavior and developing effective treatment strategies.

### 3.6 Temperature distribution in the breast tissues and tumors: effects of exercise intensities

**Fig. 9:**
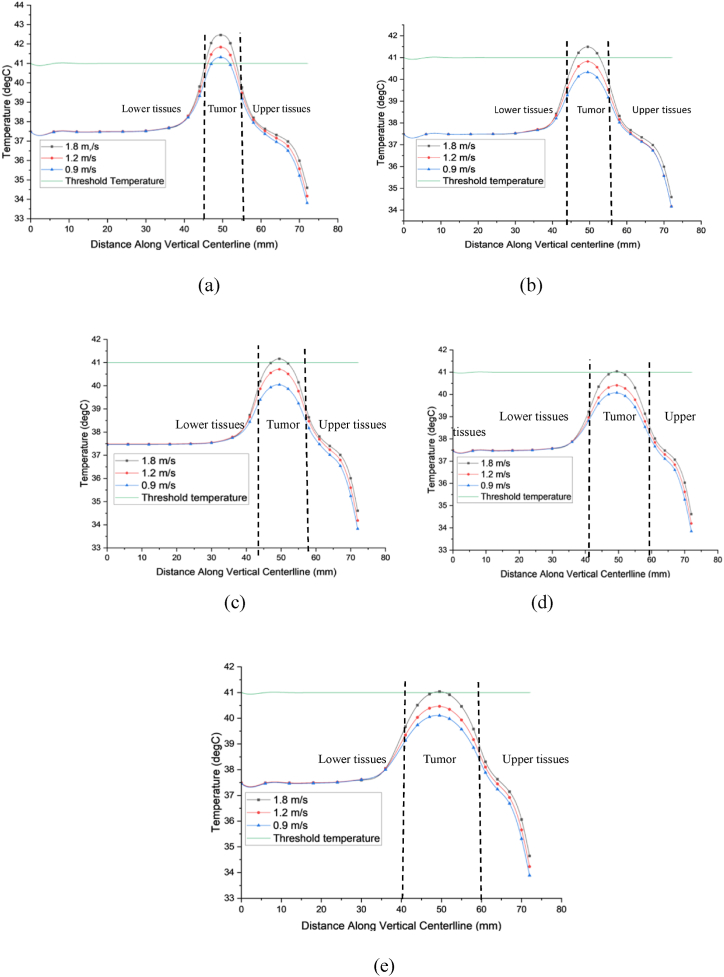
Shows how temperature varies along the vertical centerline of tumors, from the rib to the skin, at different activity levels. The analysis is presented for tumors with diameters of (a) 10, (b) 12.5, (c) 15, (d) 17.5, and (e) 20 mm.

With the ability to target and eradicate malignant tumors within a precise temperature range of 40–46 °C, thermal therapy has become a viable tactic in the fight against cancer [17,52]. Precision in temperature control is paramount to accurately replicating the thermal conditions of human body tissues, ensuring the effective elimination of cancerous growths while minimizing damage to healthy tissue, which is highly sensitive to temperature fluctuations. Specifically, the temperature within cancerous tissue should ideally be elevated to 41–45 °C, whereas in nearby healthy tissue, it cannot be higher than 41 °C [53]. Hyperthermia involves elevating the temperature of cancerous tissue to between 40 and 43 °C. It is common practice to use this method with chemotherapy and radiation therapy for cancer. This study investigating the temperature dynamics in breast tumors of varying diameters (10, 12.5, 15, 17.5, and 20 mm) during a 30-min treadmill exercise at speeds of 1.8, 1.2, and 0.9 m/s provides valuable perspectives on the possible benefits of physical activity as a therapeutic approach for breast tumors. The study's findings, depicted in Fig. 8, illustrate the induced temperature changes within tumors and the surrounding healthy tissues across different exercise intensities. Fig. 9(a) illustrates the temperature variations within a 10 mm tumor exposed to exercise speeds of 1.8, 1.2, and 0.9 m/s. The induced temperatures range from 41 to 42.92 °C, 41–42.5 °C, and 40.5–41.4 °C, respectively, increasing from the tumor's edge towards its core. In contrast, the surrounding healthy tissues maintain temperatures between 37.5 and 41 °C. Similarly, Fig. 9(b) depicts temperature changes in a 12.5 mm tumor under the same exercise speeds, showing induced temperatures ranging from 40.5 to 41.81 °C, 40.3–40.93 °C, and 40–40.5 °C, progressing inward from the tumor periphery. Healthy tissues exhibit temperatures ranging from 37.5 to 40.5 °C.

Moving to Fig. 9(c)–a 15-mm tumor subjected to identical exercise speeds records induced temperatures ranging from 40.18 to 41.22 °C, 40–40.74 °C, and 39.45–40.13 °C, again increasing from the tumor periphery to its center. Surrounding healthy tissue temperatures range from 37.5 to 40.18 °C. In Fig. 9(d), for a 17.5 mm tumor, induced temperatures range from 39.5 to 41.03 °C, 39.2–40.52 °C, and 39.18–40.11 °C, following a similar pattern from the tumor's edge towards its center. Healthy tissue temperatures surrounding this tumor range from 37.5 to 39.5 °C.

Lastly, in Fig. 9(e)–a 20-mm tumor shows induced temperatures ranging from 39.42 to 41.03 °C, 39.16–40.47 °C, and 39.15–40.09 °C, with a progression from the periphery to the center, while surrounding healthy tissue temperatures range from 37.5 to 39.42 °C. An intriguing observation from the study is the sudden temperature rise in healthy tissues near the interface with the tumor which the influence of tumor to its surrounding healthy tissues during physical activities. Despite this, the results indicate that the induced temperature within a 10 mm tumor consistently surpasses the critical threshold for tumor elimination (41 °C) during the 30-min exercise, while temperatures in surrounding healthy tissues remain below this threshold and remain stable, suggesting minimal risk of damage to healthy tissue. Importantly, the temperature increase in healthy tissue at these exercise intensities is unlikely to cause cell damage. Interestingly, the study found that physical exercise can potentially serve as a therapeutic approach for breast tumors without causing harm to healthy tissues, especially for smaller tumors. Higher exercise intensities, such as walking at a speed of 1.8 m/s, show effectiveness in treating larger tumors, whereas lower speeds demonstrate lesser impact. These findings highlight a positive relationship between exercise intensity and therapeutic efficacy, with physical exercise showing greater effectiveness in preventing tumor development and eliminating existing tumors, particularly in their early stages.

### 3.7 The most effective exercise intensities to eliminate tumor cells

**Fig. 10:**
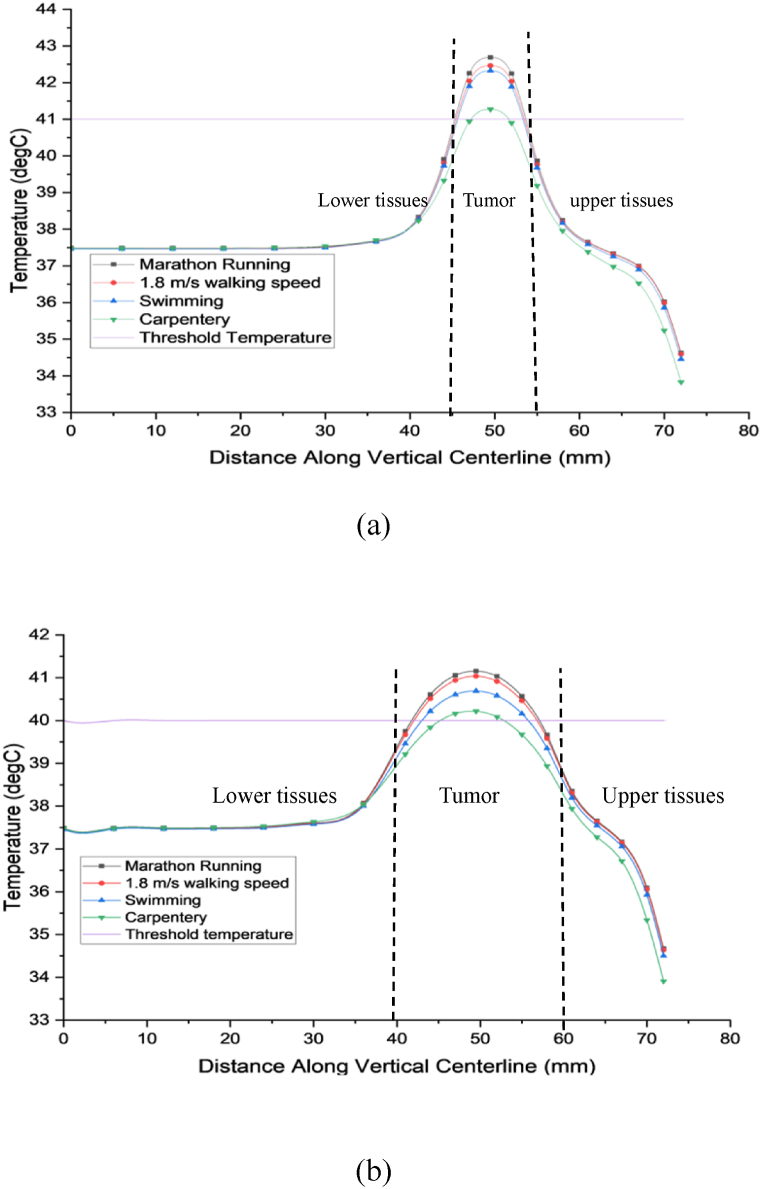
(a) illustrates a breast tumor with a 10 mm diameter, while (b) depicts a breast tumor with a 20 mm diameter.

In Fig. 10(a)–a comprehensive analysis of temperature distribution within both the breast tissue and tumor is presented across a spectrum of physical activities. These activities include carpentry, swimming, walking at a speed of 1.8 m/s, and marathon running. The data highlights the temperature responses in a 10 mm tumor, ranging from 39.76 to 41.4 °C for carpentry, 40.86–42.65 °C for swimming, 41–42.92 °C for walking, and 41–43.2 °C for marathon running, progressing from the periphery to the center of the tumor. Concurrently, surrounding healthy tissues maintain temperatures within 41 °C.

These findings reveal that the temperature within the tumor consistently surpasses the critical threshold of 41 °C necessary for the tumor elimination. Conversely, the temperature of the healthy tissues surrounding the tumor stabilizes at approximately 41 °C within 30 min of exercise, posing minimal risk of cellular damage. Consequently, physical exercises emerge as a promising therapeutic modality for tumors, as they effectively elevate tumor temperatures without jeopardizing neighboring healthy tissues. Notably, marathon running demonstrates the most significant temperature increase, reaching 43.2 °C, well within the optimal range for tumor cell eradication compared to the other exercises examined.

In Fig. 10(b)–a similar investigation is conducted on a larger 20-mm tumor exposed to identical physical activities. The induced temperatures range from 39.12 to 40.4 °C for carpentry, 39.4–41.0 °C for swimming, 39.5–41.03 °C for walking, and 39.53–41.12 °C for marathon running, progressing from the tumor periphery to its center. Surrounding healthy tissue temperatures range from 37.5 to 39.53 °C.

Moreover, the study indicates that these exercises have the potential to impede the growth of larger tumor cells, as temperatures exceeding 40 °C are observed within the larger tumors. However, the impact may not be significantly pronounced. Therefore, individuals incorporating high-intensity exercise into their routines can reduce the likelihood of succumbing to cancer. These findings underscore the significance of physical workout in cancer management, offering insights into its potential as an adjunctive therapy to conventional treatments. Moreover, the nuanced temperature responses observed in varying tumor sizes emphasize the need for tailored exercise regimens in cancer care.

### 3.8 Blood Perfusion's impact on rising tumor temperature

**Fig. 11:**
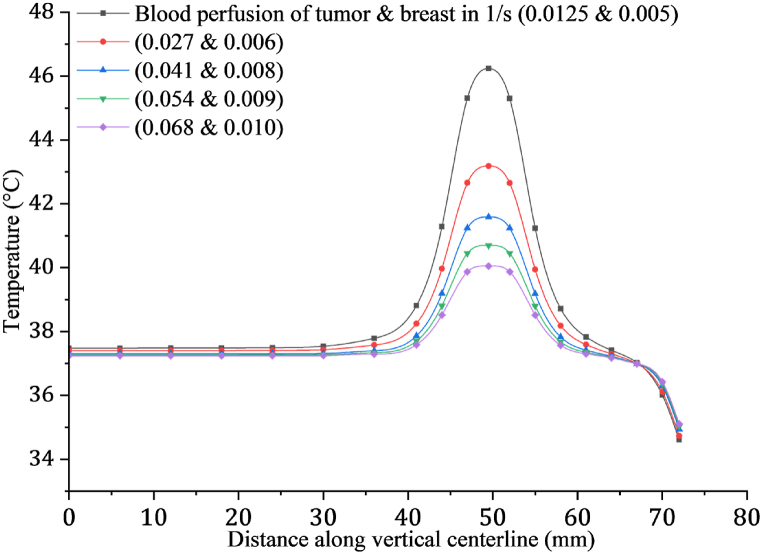
Depicts the temperature diffusion in breast and tumor at a 1.8 m/s walking speed, considering different blood perfusion rates in a tumor with a 20 mm diameter.

Our research delved into what impact blood perfusion has on heat generation within both healthy and malignant tissues during physical activity. To investigate this, we employed constant metabolic heat production rates for healthy and cancerous tissues while simulating a 1.8 m/s walking speed. The metabolic heat rates associated with this activity were 9611 $W/m^{3}$ for healthy tissues and 119786.3 $W/m^{3}$ for a 20 mm tumor. We conducted our analysis by considering various blood perfusion rates within the ranges outlined by Camilleri [45]. For tumor tissue, the blood perfusion rate ranged from 0.00129 to 0.005 $s^{-1}$, while for normal breast tissue, it ranged from 0.00045 to 0.00093 $s^{-1}$. Within these ranges, we selected specific rates in our study for 5 steps: 0.0125, 0.027, 0.041, 0.054, and 0.068 $s^{-1}$ for malignant tissue, and 0.005, 0.006, 0.008, 0.009, and 0.01 $s^{-1}$ for healthy tissue. Fig. 11 presents the temperature increase observed in both breast tissue and a 20-mm tumor while walking at the specified speed. Our findings illustrated that even minor fluctuations in blood perfusion have a pronounced impact on temperature variation. Particularly noteworthy was the observation that reducing blood perfusion while maintaining constant metabolic heat led to a significant rise in both healthy tissue and tumor temperature.

This study underscores the importance of considering blood perfusion dynamics in understanding thermal changes within tissues during physical activity. The results suggest that alterations in blood perfusion rates, even within clinically feasible ranges, can markedly affect the thermal response of tumors, which may have implications for treatments involving thermal modulation of cancerous tissues.

### 3.9 Temperature rise during exercise: determining the ideal duration for physical activity

**Fig. 12:**
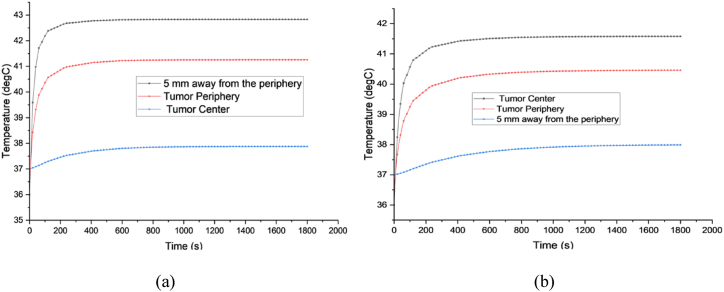
Shows the temperature increase at various locations in both the breast and the tumor at two different walking speeds: (a) 1.8 m/s and (b) 0.9 m/s.

Fig. 12(a) and (b) illustrate the temperature elevation at various positions within the breast and tumor over time as an individual walks on a treadmill at 1.8 and 0.9 m/s speeds. The tumor, having a diameter of 10 mm, is assessed at three points along the vertical centerline: (1) the tumor center, (2) the tumor periphery, and (3) a location 5 mm below the tumor periphery. After 20 min of treadmill activity at both speeds, the temperature at the third point stabilizes at 38.2 °C, remaining below the human body's threshold for hyperthermia. At the second point, temperatures rise to above 41 and 40.5 °C for walking speeds of 1.8 and 0.9 m/s, respectively, after 20 min, and then stabilize. These temperatures can hardly cause damage to body cells, but the temperature in the tumor can selectively eliminate only the tumor cells. The first point experiences temperature increases to 42.83 and 41.58 °C for walking speeds of 1.8 and 0.9 m/s, respectively, after 20 min, and stabilizes thereafter. These elevated temperatures are expected to induce damage specifically to tumor cells situated in the tumor. Notably, the body temperature undergoes a rapid increase for the first 5 min and then it becomes slow for a further 15 min during exercise, after which it stabilizes. Therefore, to optimize results, individuals should aim to sustain high-intensity exercise for at least 20 min.

## 4 Assumptions and limitations

The assumption of tissue uniformity may not fully capture the variations in tissue properties within the breast, affecting the accuracy of simulation results. No external heat is applied to tissues, so Q is assumed to be zero in the governing equation. Based on previous studies, it is assumed that the metabolic heat and blood perfusion rates of the breast and tumor increase linearly with exercise intensities. Restricted generalizability due to individual variations in breast tissue composition, metabolism, and response to physical activity. The study could not investigate the sensitivity analysis because it used an average model of breasts irrespective of age, height, age, and size. No experimental or computational studies on physical exercise, tumor prevention and heat generation during physical activities exist. So, the present study was validated for resting conditions. The absence of validation against clinical data limits the generalizability and applicability of the simulation results to real-world scenarios.

## 5 Conclusions

The motive of this research was to look at the possibility of using physical activity as a type of thermal therapy and elucidate the underlying mechanisms involved. Employing a 3D model representing the breast and tumor, the research observed that cancerous tissues (tumors) exhibited a significantly higher heat generation than adjacent normal tissues at rest and during physical activities. As tumors increased in size, a concentric contraction of the cold area was noted inside the breast. The study underscored that smaller-diameter tumors manifested elevated temperatures at rest and during exercise, highlighting the pivotal role of tumor growth and size in heat generation inside the tumors. The temperature range within tumors of different sizes ranged from 41 to 43.2 °C, while healthy tissue did not exceed 41 °C during various exercises like marathon running, swimming, carpentry, and treadmill walking at speeds of 1.8 m/s. The exercise intensity positively correlated with temperature increase in both healthy and cancerous tissues. Both the tumor's and healthy tissues' temperatures rise noticeably when a steady metabolic heat is maintained while blood perfusion is reduced. The research also identified the ideal time for high-intensity exercise to yield the best results. It recommends that people should participate in high-intensity physical activities for a minimum of 20 min to enhance therapeutic effects. The findings concluded that physical exercises could serve as a thermal ablation therapy for tumors, with higher-intensity exercises proving more effective. In summary, the research advocates for enhanced physical activity to prevent cancer, promote a healthy lifestyle, and contribute to longevity.

## Ethics declarations

In developing and publishing our computational model, we affirm ethical practices. We ensure informed consent, data integrity, impartiality, responsible technology use, regulatory compliance, acknowledgment of contributions, conflict disclosure, and ongoing ethical review. We were signed by the authors, with a commitment to ethical research standards.

## Funding

This research did not receive any specific grant from funding agencies in the public, commercial, or not-for-profit sectors.

## Data availability

Data will be made available on request.

## CRediT authorship contribution statement

**Mohammad Junaid:** Writing – review & editing, Writing – original draft, Visualization, Validation, Resources, Project administration, Methodology, Investigation, Formal analysis, Data curation, Conceptualization, Software. **Abul Mukid Mohammad Mukaddes:** Writing – review & editing, Visualization, Supervision, Investigation, Formal analysis. **Md. Mahmud-Or-Rashid:** Writing – review & editing, Supervision, Investigation, Data curation, Project administration.

## Declaration of competing interest

The authors declare that they have no known competing financial interests or personal relationships that could have appeared to influence the work reported in this paper.
